# Co-Adsorption of Novobiocin and Three Common Surfactants on Polystyrene: Molecular Insights from Experiments and Simulations

**DOI:** 10.1021/acs.langmuir.6c02613

**Published:** 2026-09-12

**Authors:** Diana S. Barrios, Gabriel D. Barbosa, Sepideh Razavi, Helen I. Zgurskaya, Brian P. Grady, Alberto Striolo

**Affiliations:** † School of Sustainable Chemical, Biological and Materials Engineering, 6187 University of Oklahoma, Norman, Oklahoma 73019, United States; ‡ Materials Science and Engineering Program, The University of Oklahoma, Norman, Oklahoma 73019, United States; § Department of Chemistry and Biochemistry, The University of Oklahoma, Norman, Oklahoma 73019, United States

## Abstract

In several environments, including wastewater treatment plants (WWTPs), plastics encounter surface-active molecules such as antibiotics and surfactants, essential components of consumer products, detergents, and drugs. In these conditions, synergistic and antagonistic effects could affect how surfactants and antibiotics compete for access to plastic surfaces. Experimental and computational results are presented here to quantify the adsorption on polystyrene (PS) of the antibiotic Novobiocin (NVB) in combination with adsorption of three surfactants—the anionic docusate sodium salt, AOT; the cationic cetyltrimethylammonium bromide, CTAB; and the nonionic hexaethylene glycol monododecyl ether, C_12_E_6_. The experimental results obtained with a quartz crystal microbalance with dissipation monitoring (QCM-D) suggest that, rather than removing NVB, the three surfactants yield an adlayer on the NVB film pre-adsorbed on PS. On the contrary, an aqueous solution of NVB partially removes pre-adsorbed surfactant films, and co-adsorption at the PS surface likely occurs. To rationalize these observations, molecular simulations were conducted at low concentrations of NVB and surfactants, somewhat similar to those found in aquatic environments. The computational results show that the interactions between NVB and PS are stronger than those between surfactants and PS. Combined, the results show that NVB and these surfactants are likely to co-adsorb on the polystyrene surface. Although generalization is premature, the observed synergism might facilitate the environmental dispersion of both antibiotics and microplastics, suggesting that advanced technologies are required to properly handle plastics, microplastics, and antibiotics in WWTPs, towards preventing the spread of antibiotic-resistant microorganisms.

## Graphical abstract

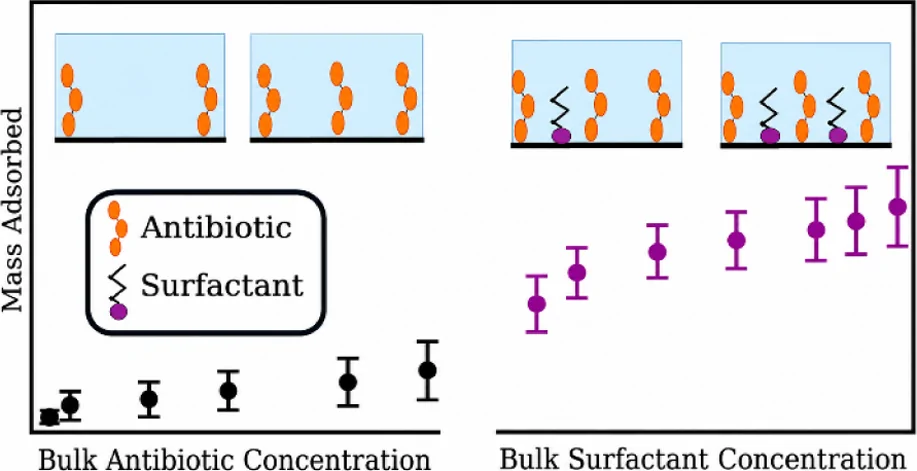

## 1 Introduction

Although significant progress has been secured towards achieving the UN Sustainable Development Goals (SDGs) by 2030,^1^ much remains to be done. For example, UN SDG number 3 has the ambition to end epidemics, while UN SDG number 6 seeks to ensure availability of clean water and sanitation for all. Unfortunately, emergent contaminants can compromise both these goals, especially when their environmental risks are compounded. A specific example is represented by the environmental dispersion of microplastics and antibiotics, both of which are emergent contaminants.

Microplastics, plastic particles of size <5 mm,^2,3^ are classified as either primary—coming from manufactured items such as pellets, beads, or fibers—or secondary—formed via degradation of plastic products.^4^ Plastics and microplastics accumulate in the environment, perhaps surpassing 12,000 Mt by 2050.^5^ Although a large variety of polymers are available to manufacture plastic products, the main ones are polyethylene, polypropylene, polyvinyl chloride, polyethylene terephthalate, and polystyrene. As a side note, only 9% of the 6300 Mt of plastics produced up to 2015 have been recycled.^6^ Films and biofilms can form on plastic surfaces, and microplastics can sink or float in water depending on the resulting surface properties, which are affected by adsorbed surface films and biofilms.^7^ Of note, Wang et al.^8^ concluded that biofilm-developed microplastics could act as vectors to introduce pollutants, as well as attached microorganisms, to aquatic environments and organisms. To assess the likelihood of this scenario, it should be mentioned that 10% of microplastics present in wastewater exit wastewater treatment plants (WWTPs) in the effluent streams.^9^

The other emergent class of contaminants considered here is represented by antibiotics, which, starting from the discovery of penicillin and its mass production, revolutionized modern medicine and are now essential for extending life expectancy, improving the quality of life, and enhancing livestock productivity, among other applications.^10,11^ However, overuse and misuse of antibiotics, via their resultant environmental accumulation, is breeding antimicrobial-resistant microorganisms (AMRs).^12–14^ Both antibiotics and antimicrobial-resistant genes have been found in rivers^15^ and clouds.^16^ Estimates indicate that, from 1990 to 2021, 4.71 million human deaths were associated with bacterial AMR,^17^ raising the alarm of a potential future AMR-related pandemic.^18^

Perhaps because microplastics can function as vectors for distributing contaminants in the environment, including antibiotics and AMRs,^19,20^ many studies have documented sorption of antibiotics on plastics, focusing on the amount adsorbed as well as kinetics. Stapleton et al.^21^ critically reviewed the literature, identifying what plastics have been studied, as well as the classes of antibiotics that have been considered. The results are important from the environmental point of view^22,23^ —correlations between antibiotics and microplastics detection in manure and farm soil have been reported^24^—as well as for packaging and administration of human drugs and biologics, and perhaps also for designing processes that use microplastics to remove antibiotics from water.^25^ Mounting data show that antibiotic adsorption on plastic surfaces depends on a variety of conditions, including (a) polymer type; (b) particle size, shape, and surface area; (c) degree of weathering; (d) solution pH and salinity; and (e) presence of competing contaminants, adsorbents, and surface-active species.^21^ Plastic aging is of particular interest, as aging can lead to surface modification, changes in the structure/microstructure of the particles, the release of radicals and other chemicals—all processes that affect the interactions between plastics and antibiotics.^26^

Ultimately, controlling antibiotic adsorption on microplastics would be possible when the molecular interactions responsible for the various phenomena just summarized are well understood, a task made challenging by the fact that realistic environments comport the presence of multiple compounds in addition to plastic surfaces and antibiotics; as a start, experiments conducted in controlled environments can elucidate the molecular interactions responsible for adsorption. One experimental technique that probes molecular films on surfaces is the quartz crystal microbalance with dissipation (QCM-D), which has been frequently used to monitor the formation or desorption of protein films as well as layer-by-layer deposited films on various substrates.^27,28^ In the QCM method, the vibration frequency of the sensing element (i.e., the quartz crystal) changes as adsorption of molecules from the bulk onto the surface increases the mass of the oscillating substrate. The technique has been widely used to quantify the adsorption of surfactants,^29–32^ as well as the competition between surfactant adsorption and that of other molecules on a given surface.^33–35^

To interpret experimental data, it is essential to have access to dissipation data, which enables the discrimination between “rigid” and “viscoelastic” films formed on the surface. Dissipation values below 2 × 10^–6^ are expected for “rigid” films, while those above this threshold correlate with “viscoelastic” films.^36^ When the films are “rigid”, the Sauerbrey model provides a good measure of the amount adsorbed;^37^ more computationally demanding models are necessary to extract the mass adsorbed when the films are viscoelastic.^38^ One of the limitations of the QCM-D method is that it is restricted to sensing elements (the quartz crystals) covered with selected materials. The experiments discussed below were conducted on quartz crystals covered by polystyrene (PS), one of the major classes of polymers used by our society. Further, QCM-D results only provide information on the mass adsorbed, without distinguishing between adsorbed solvent vs. the molecule(s) of interest. In the case of surfactant adsorption, this challenge has led to significant debate;^39,40^ it is now accepted that QCM-D data can include large amount of solvent, trapped within viscoelastic films, while rigid films are assumed to be mostly free of solvent. When multiple surface-active species are present, QCM-D data only provide information on the total mass adsorbed, without distinguishing the various components.^31,33–35^ To provide a molecular-level interpretation of the experiments, it is possible to conduct molecular dynamics simulations (MD). For example, it has recently been suggested that the high adsorbed amount measured for AOT surfactants on PS is consistent with the formation of interconnected multilayered films with large amounts of trapped water, while the low amount of CTAB adsorbed on PS is consistent with a loose adsorbed monolayer.^41^ Repulsions between CTAB surfactant headgroups, coupled with weak substrate-surfactant attractions, were found responsible for these observations.^41^

Building on those prior results, experiments conducted with a QCM-D are coupled here with atomistic MD simulations to investigate potential competition between antibiotics and surfactant adsorption on PS. The experimental adsorption isotherms previously measured for AOT, C_12_E_6_, and CTAB surfactants^41^ were used as a baseline. Novobiocin (NVB), an aminocoumarin antibiotic featuring an aromatic core and a carbohydrate substituent, with molecular weight 612.6 g/mol, was chosen to investigate its adsorption capabilities. Of note, the molecular structure of NVB presents several aromatic rings, which should facilitate attractions with the PS substrate.^42^ In Figure 1, the molecular structures of PS, NVB, AOT, C_12_E_6_, and CTAB are shown. The locations of partial positive and negative charges are shown to highlight potential mechanisms that could drive adsorption.

**Figure 1:**
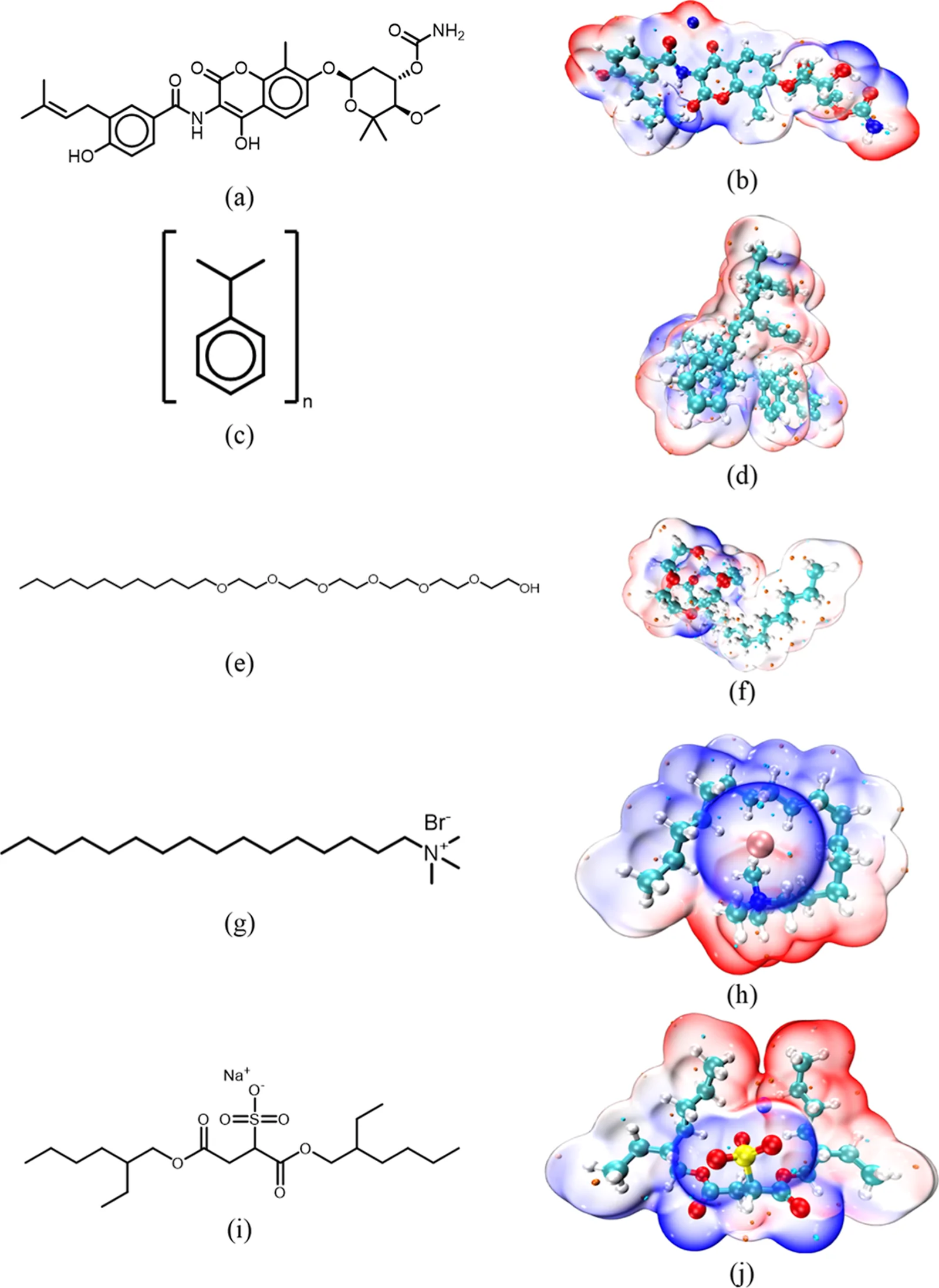
Molecular structure (left) and charge densities (right) of, from top to bottom, NVB, a PS monomer, C_12_E_6_, CTAB, and AOT. Electrostatic surface potentials (ESP) are also shown where local extrema are indicated by small spheres using the following color scheme: cyan for local minima and orange for local maxima. ESP calculations were carried out using the methodology described elsewhere.^43^

The experimental results, corroborated by MD simulations, show that NVB is strongly adsorbed on PS, and all three surfactants adsorb alongside the pre-adsorbed NVB molecules on PS. While these results are too few to enable generalization, they demonstrate that a fundamental investigation on the antibiotic adsorption on plastics is feasible, and that molecular-level details determine the properties of the adsorbed films.

## 2 Materials and Methods

### 2.1 Materials

Three common surfactants, purchased from Sigma-Aldrich, were used for this study: the nonionic hexaethylene glycol monododecyl ether (C_12_E_6_, ≥98.0% purity), and the ionic docusate sodium salt (AOT, ≥99.0% purity) and cetyltrimethylammonium bromide (CTAB, ∼99% purity). While C_12_E_6_ and AOT were used as received, CTAB was recrystallized 3 times in an acetone-ethanol solution (10:1 v/v) before use.^44^ To confirm the purity of the surfactants, their critical micelle concentrations (CMCs) were measured using the Wilhelmy plate approach, as detailed in our previous study;^41^ the CMCs were 0.075 ± 0.05 mM for C_12_E_6_, 0.982 ± 0.69 mM for CTAB, and 2.78 ± 0.82 mM for AOT, which are consistent with literature values of approximately 0.07 mM for C_12_E_6_,^45^ 0.9 mM for CTAB,^46^ and 2.81 mM for AOT.^47^

Novobiocin (NVB) was also purchased from Sigma-Aldrich and used as received.

### 2.2 Quartz Crystal Microbalance with Dissipation

Adsorption experiments were performed using a QCM-D, originally purchased from Q-Sense Analyzer (now Biolin Scientific). All experimental details are described in our recent work^41^ and briefly outlined here. To monitor adsorbed mass, the instrument monitors the oscillation frequency of a quartz crystal, purchased from Biolin Scientific, which has a nominal resonance frequency of 5 MHz. The sensing elements, covered by PS by the manufacturer, were identified as PS-hydrophobic polystyrene, QSX 305. The sensing elements were characterized by using the atomic force microscope (AFM), to quantify surface roughness and potential irregularities, and X-ray photoelectron spectroscopy (XPS), to confirm the chemical composition of the surface. Results are described in our recent report^41^ and reproduced in Figure 2. Figure 2a andFigure [Fig fig2]b show the topographic image of a pristine polystyrene sensor surface and the corresponding line scan profile. The polystyrene substrate exhibits depressions as low as 6 nm combined with peaks of 4 nm with a root mean roughness (RMS) of 1.61 nm, which is less than the RMS of the standard gold sensing elements. XPS shows that the pristine polystyrene sensor is mostly polymeric with negligible amounts of gold, oxygen, and nitrogen.

**Figure 2:**
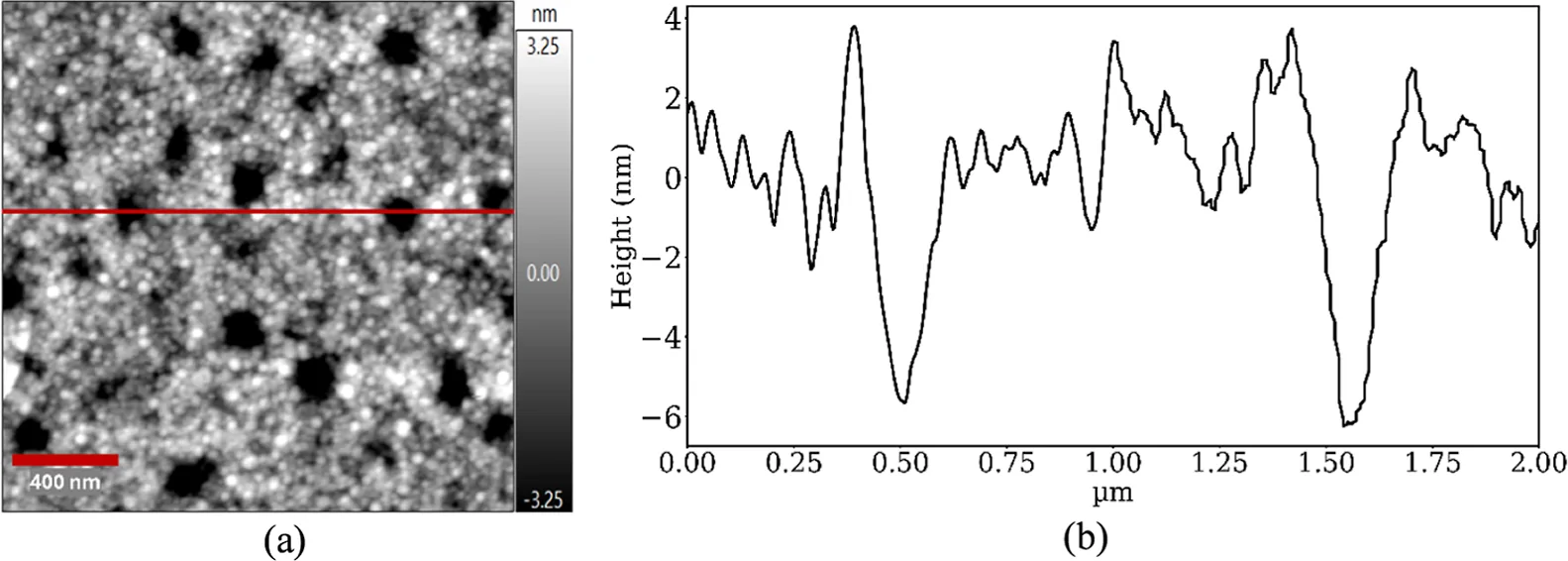
(a) AFM topographic image of a pristine polystyrene sensor cleaned by 10 mM of CTAB and Milli-Q water over a surface of 2 × 2 μm^2^ area and (b) the corresponding line profile analysis. Reproduced with permission from ref 41. Copyright [2026] [Elsevier].

To estimate the mass adsorbed, the Sauerbrey model can be employed when the dissipation is less than 2 × 10^–6^, as discussed in the introduction. For the systems considered here, the dissipation is within this limit except for the experiments conducted using AOT, in which case the dissipation is higher than 2 × 10^–6^. Hence, in most cases, dissipation values confirm that the Sauerbrey model provides a reasonable approximation of the adsorbed amount. Exceptions are discussed in the text. As stated previously, the QCM-D data show the total amount adsorbed, which, in the present experiments, could include the combination of surfactant, antibiotics, and solvent. The experiments were conducted in Milli-Q water (18 MΩ cm). The pH was not adjusted and remained between the reported pKa values of NVB (4.3 and 9.5).^48^ No electrolytes were added to the systems other than those initially present in the surface-active species used for the experiments.

### 2.3 Adsorption Isotherm

Stock solutions in Milli-Q water at 10 times CMC were prepared for surfactants; the stock solution of NVB was prepared at 0.5 g/L in Milli-Q water. For NVB, we did not attempt to measure a CMC, as it was assumed that this antibiotic does not form micelles. The stock solutions were subsequently diluted to the desired concentrations before adsorption isotherm experiments.

The procedure, detailed in our previous work,^41^ was followed to clean the sensors by repeatedly flushing them with 10 mM CTAB solution followed by Milli-Q water, establish a stable baseline and ensure that each measurement reached steady state. Because of significant sensor-to-sensor variations in roughness, even for nominally identical QCM sensors, all measurements presented below were obtained from the same polystyrene-coated quartz crystal. Each adsorption isotherm was repeated at least three times to ensure reproducibility. The reported results are the mean and standard deviation at each concentration. To ensure that cleaning procedures fully removed all adsorbed mass and that the sensors did not change surface features during the course of an experimental campaign, C_12_E_6_ isotherms were periodically remeasured and compared with the isotherm obtained when the sensors were first used.

During the adsorption experiments, the sensing elements were exposed sequentially to aqueous solutions containing either surfactants or antibiotics at different concentrations. Two experimental sequences were implemented:

(A) First the NVB film was formed, measuring an adsorption isotherm at increasing NVB concentration from 0.01 to 1 mg/L, followed by a surfactant adsorption isotherm for one of the surfactants in the concentration range 0.1–1 C/CMC. In the case of surfactants, the bulk concentrations reported in the experimental plots were normalized by the CMC to account for differences in surfactant activity and adsorption behavior. Because the CMC is different for the three surfactants, the maximum concentration used ranged from 0.007 mM for C_12_E_6_ to 2.81 mM for AOT.
(B) The surfactant film was formed first by increasing the concentrations from 0.1 to 1 C/CMC, followed by exposure to NVB aqueous solutions from 0.01 to 1 mg/L.

The bulk NVB concentration was maintained at 1 mg/L or below to consider concentrations detectable by QCM-D while avoiding very large concentrations, as concentrations examined in this study already exceed those observed in wastewater treatment plants.^49^ These low concentrations led to comparatively large uncertainties, but allowed the investigation of concentration ranges close to those expected in WWTPs. Previous studies that investigated antibiotic adsorption onto microplastic using plastic particles have commonly used concentrations equal or greater than those examined in this work.^50–52^

Comparing the results from the two sequences assessed whether the order of exposure affects the adsorbed amount. It should be stressed, however, that the polystyrene substrates are exposed to bulk solutions containing pure components, as NVB solutions do not contain surfactants, and, vice versa, the surfactant bulk solutions do not contain NVB.

### 2.4 Molecular Simulations

To help interpreted experiments, atomistic molecular dynamics simulations (MD) were conducted. All simulations were performed with the software package GROMACS 2023.3,^53^ implementing the simulation protocol described previously^41^ and briefly described here. Each simulated system was equilibrated for 300 ns at ambient conditions; once equilibration was achieved, additional 500 ns of simulations were conducted and used for production.

Nonbonded interactions were treated with a 1.0 nm cutoff for both Lennard–Jones and electrostatic interactions, consistent with the OPLS-AA force field.^54–57^ The force field parameters implemented were obtained from the LigParGen Web server. The OPLS-AA force field was chosen for the present study because it demonstrated reliable predictions for structural, thermodynamic, and mechanical properties of polymeric systems.^58–61^ This model has also been widely used to model the thermodynamic behavior of nonionic ethoxylated surfactants,^62–65^ as well as AOT^66^ and CTAB.^67^ Recent benchmarking studies have shown that OPLS-AA provides a suitable and computationally effective description of AOT-containing systems.^66^ Long-range corrections for the electrostatic interactions were implemented using the particle mesh Ewald (PME) method.^68^ Periodic boundary conditions were applied in all three spatial directions, although the solid–liquid interface prevents the formation of 3D bulk systems. To ensure reproducibility and minimize bias from the initial configuration, all simulations were performed in triplicate using independent replicas.

Initial configurations were generated using the PACKMOL software.^68^ As shown in Figure 3, molecular simulations were initiated from a film of surfactants and antibiotics deposited on a PS substrate.

**Figure 3:**
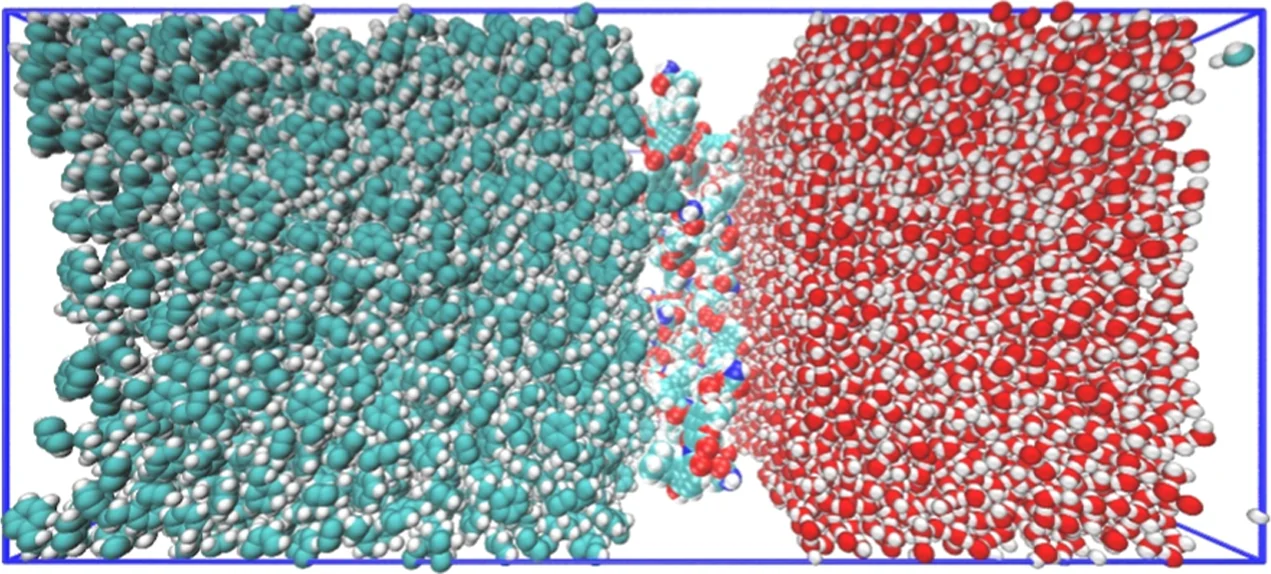
Initial configuration of NVB in the PS/water interface (7.5 × 7.5 × 17.0 nm^3^). Color code: cyan—carbon; red—oxygen; white—hydrogen; blue—nitrogen.

The PS substrate was modeled using chains composed of 80 repeat units. To construct the substrate, 40 PS molecules were randomly placed in a simulation box and energy-minimized using the steepest descent algorithm. The system was then equilibrated in the canonical (NVT) ensemble for 10 ns, followed by further equilibration in the isothermal–isobaric (*NPT*) ensemble for 100 ns. Convergence toward equilibrium was assessed by monitoring the potential energy, density, and box dimensions. Temperature and pressure were controlled using the velocity-rescaling thermostat and the Parrinello–Rahman barostat, with time constants of 1 and 5 ps, respectively.

The surfactant/antibiotic film was constructed by depositing the desired number of molecules near the PS surface, followed by solvation with water. Surfactants and NVB molecules were deposited in contact with the PS substrate because the process of adsorption/desorption occurs experimentally in time-scales of the order of microns, which are not yet accessible to atomistic MD simulations such as those used in the present study. Placing the various components on the substrate allows for the investigation of the film structure. Maintaining low surfactant concentrations is meant to prevent the observation of long-lived metastable states. After an initial energy minimization using the steepest descent method, the system was equilibrated for 10 ns in the *NVT* ensemble and subsequently for 200 ns in the *NPT* ensemble. These equilibration stages were sufficient to stabilize the systems, as confirmed by monitoring the potential energy and density profiles. Production simulations of 500 ns were then performed to collect structural properties. All simulations were conducted in three independent replicas to reduce bias associated with the initial configurations.

The final configuration obtained from the simulations of aqueous NVB on PS was used to construct systems also containing surfactants. Specifically, water molecules were removed; surfactant molecules were inserted at the desired surface density and then water molecules were added to fill the empty space. These systems contained 10,000 water and 20 surfactant molecules. Notably, all simulations were performed under charge-neutral conditions using the appropriate counterions: sodium ions for AOT, bromide ions for CTAB, and sodium ions to neutralize the net negative charge of NVB. The surface area of the PS substrate was of 7.5 × 7.5 nm^2^. The surfactant surface densities considered in the simulations are consistent with experimental data under the assumption that the surface is smooth (23 ng of surfactants per cm^2^ of PS).

The NVB-PS association was quantified using a heavy-atom contact criterion. An NVB molecule was classified as directly contacting the PS substrate when at least one of its heavy atoms was within 0.70 nm of a PS heavy atom. The time-dependent NVB-PS contact number was calculated using the same distance cutoff. For NVB molecules in contact with PS, the interfacial orientation was characterized using the vector connecting atoms C0I, the aromatic carbon bonded to the amino group, and C0C, the aromatic carbon bonded to the oxygen-containing saturated ring. This vector defines an axis spanning two connected aromatic regions of NVB. Local PS surface atoms were identified using the ITIM^68^ algorithm as implemented in Pytim.^68^ For each NVB molecule in contact with PS, a local surface plane was fitted by principal-component analysis conducted on the 30 nearest interfacial PS atoms, accounting for periodic boundary conditions; the eigenvector associated with the smallest variance was taken as the local surface normal. The angle θ between the C0I–C0C vector and the local PS surface normal was then calculated and reported as |cos θ|.

## 3 Results and Discussion

### 3.1 Adsorption of NVB on Polystyrene

In Figure 4, the adsorption isotherm measured for the PS substrate exposed to aqueous solutions of NVB is shown. Figure 4a presents the adsorbed amount, as estimated using the Sauerbrey relation, while Figure 4b shows the dissipation. The dissipation data confirm that the Sauerbrey model is acceptable to use for estimating the mass adsorbed for NVB on PS. As the concentration of NVB increases, the mass adsorbed slightly increases, reaching ∼27 ng/cm^2^. It is noted that the uncertainties in these measurements are significant, such that the amount of NVB adsorbed can be considered statistically constant when the NVB concentration in bulk water is above 0.5 mg/L.

**Figure 4:**
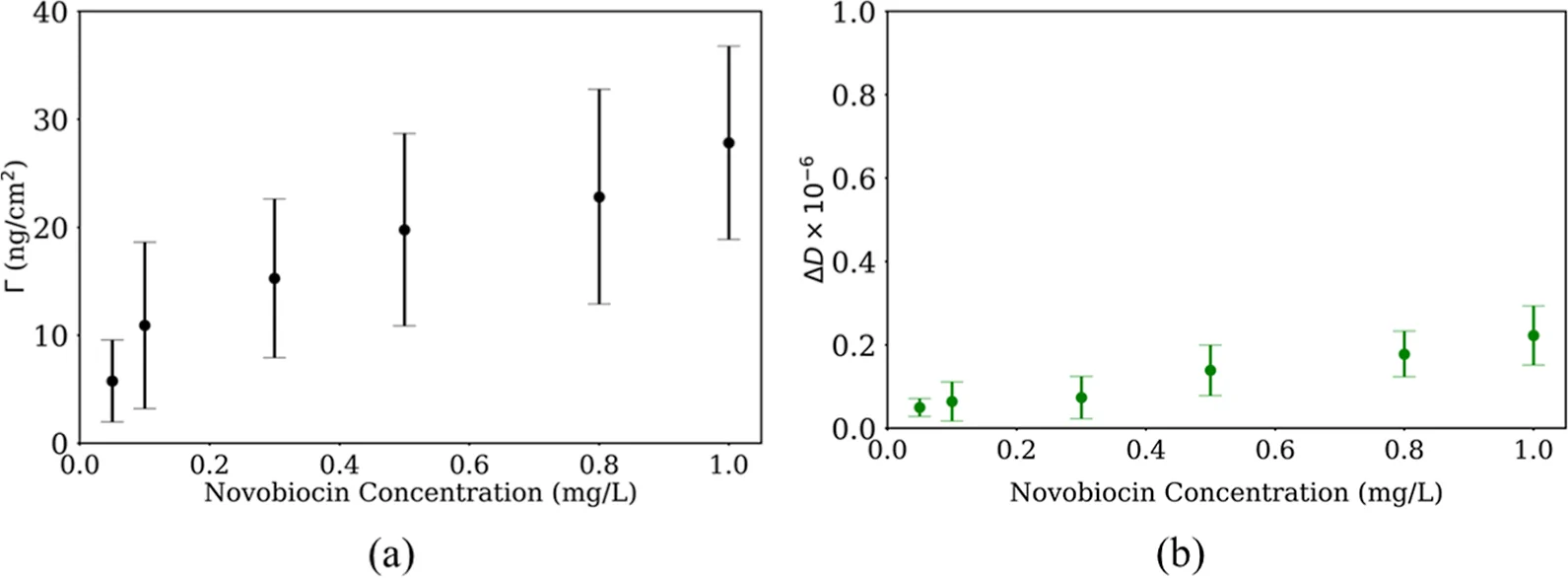
Experimental adsorption isotherm (a) and change of dissipation (b) of Novobiocin from 0.05 mg/L to 1 mg/L. Experiments were conducted with the QCM-D. During the experiments the temperature was maintained at 298 K.

To our knowledge, there are no previous studies that quantify the mass of antibiotics absorbed onto polymer-coated QCM-D crystals. Values obtained here cannot be directly compared with results from other methods because the PS QCM-D substrate is substantially smoother than the plastic particles used in previous works based on SEM imaging characterization.^50–52^ Nevertheless, the literature consistently shows that antibiotic absorption onto plastics is governed by specific intermolecular interactions.^42,69–72^ One factor dictating the adsorption capacity onto PS is the hydrophobicity of the antibiotic. Most antibiotics are amphiphilic and vary by their low 1-octanol-water partition coefficient (log *P*_o/w_). Those with higher log *P*_o/w_ values tend to exhibit stronger adsorption onto PS.^70,73^ Novobiocin has a comparatively high log *P*_o/w_ of 2.9;^25,74^ its aromatic rings further enhance its affinity for PS through π–π interactions.^68^

It is useful to compare the amount of NVB adsorbed on PS to that of the pure surfactants adsorbed on the same PS. In our previous work, we reported the adsorption of C_12_E_6_, CTAB, and AOT on PS at *T* = 298 K.^41^ This comparison should be made with caution because a plateau is not clearly shown in Figure 4a, whereas the surfactant isotherms showed levelling of the adsorbed amount. The adsorbed amount of surfactant onto the PS substrate, estimated using the Sauerbrey relation for the isotherms of C_12_E_6_ and CTAB and the Voigt viscoelastic model for AOT—as discussed in our prior work, exceeded the NVB mass adsorbed. C_12_E_6_ reached approximately 150 ng/cm^2^, CTAB plateaued near 48 ng/cm^2^, and AOT reached a maximum of about 670 ng/cm^2^. The latter value likely includes trapped water, while for the other two surfactants, the observed plateau may reflect saturation in the limitation in interfacial packing on the PS surface. Within the concentration range examined, AOT and C_12_E_6_ adsorbed larger amounts than NVB; the amount of CTAB adsorbed was of similar magnitude compared to NVB at the conditions considered in the present manuscript.

Molecular dynamics (MD) simulations offer a complementary viewpoint to analyze NVB-PS interactions as well as the structure of NVB-surfactant films formed on PS. The results from simulated trajectories were quantified in terms of density profiles along the direction perpendicular to the PS substrate, as shown in Figure 5. Distinct bulk phases are observed for PS and water; moving away from the interface, the water density increases from zero to its bulk value, while the PS density decreases to zero over a shorter length scale moving from the PS substrate to bulk water. NVB localizes at the water-PS interface, consistent with persistent adsorption under the simulated conditions. The length scale over which the density changes is roughly the same for water and NVB. Notably, a small peak in the NVB density profile appears at 8.5 nm, consistent with the presence of NVB molecules above the first interfacial layer, as also observed via the visualization of the simulation trajectories.

**Figure 5:**
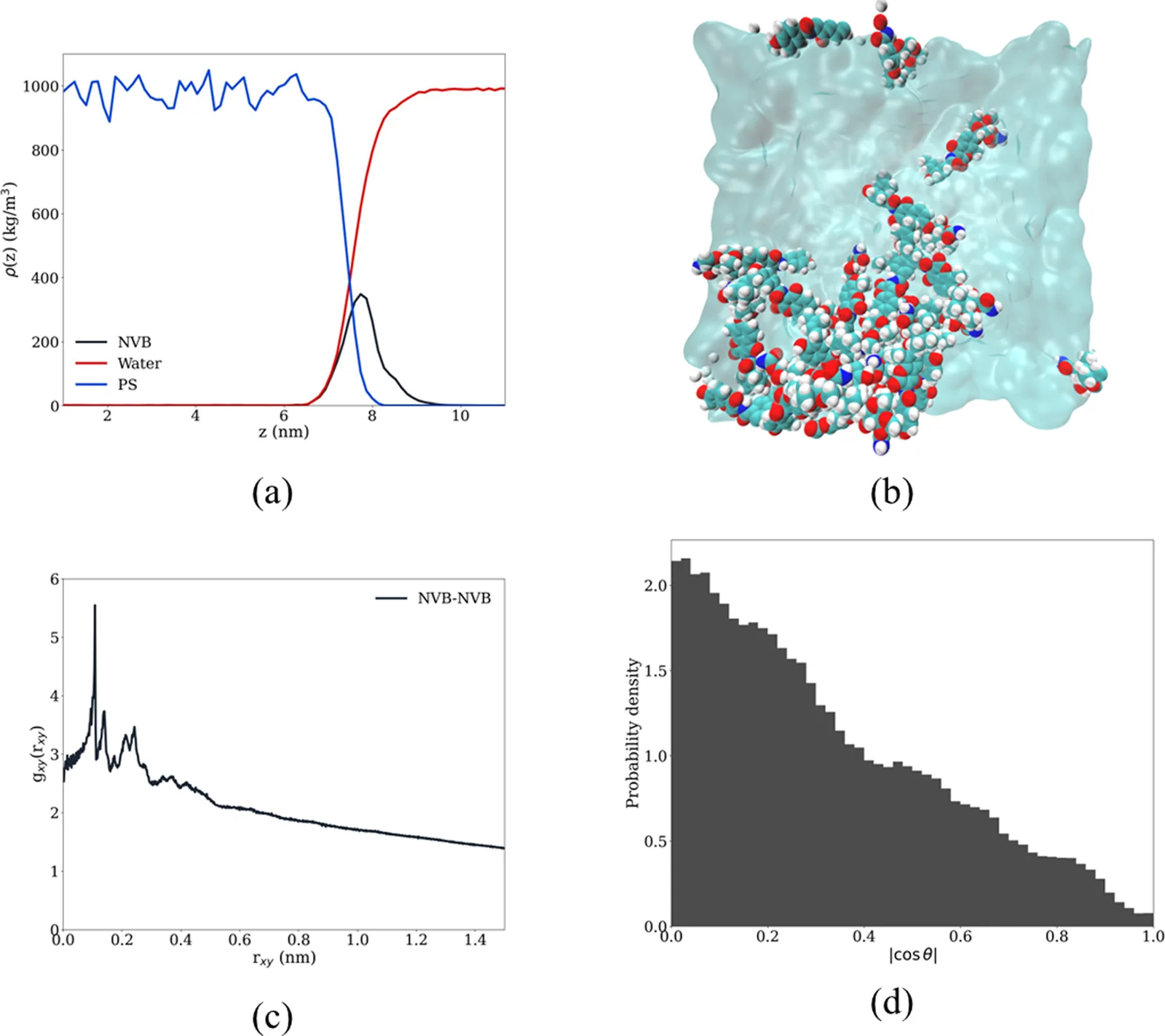
(a) Density profiles along the *z*-direction, perpendicular to the PS-liquid interface, for polystyrene (PS), novobiocin (NVB), and water in the PS/NVB/water system. All atoms of NVB are used to obtain the density profiles. (b) Representative simulation snapshot, providing the top view of NVB molecules adsorbed onto the PS substrate. Water molecules are not shown for clarity. (c) In-plane radial distribution functions (*g*_*xy*_(*r*)) between NVB molecules within a plane parallel to the PS substrate. (d) Orientation distribution of NVB molecules in contact with PS, expressed as the probability density of |cos θ|. In (d), θ is the angle between inter-aromatic NVB axis and the local PS surface normal. The simulations were conducted at 298 K and 1 bar.

The representative snapshot in Figure 5b illustrates NVB molecules localized and laterally associated at the PS surface, consistent with the density profile and radial distribution functions (RDF) analysis, commented below. This structure is consistent with the electrostatic potential (ESP) maps shown in Figure 1, where NVB exhibits a more heterogeneous, interactive charge distribution than PS, whose surface is electrostatically uniform. The polarity of NVB promotes intermolecular electrostatic interactions and self-association on the PS surface. To obtain more quantitative comparisons, general interaction property functions (GIPFs) obtained from density functional theory calculations are summarized in Supporting Information, Table S1. The descriptors include molecular volume (*V*_m_), minimum and maximum electrostatic surface potential (ESP) values, surface area (SA), average positive and negative ESP values ( ${\mathrm{V̅}}^{+}$ and ${\mathrm{V̅}}^{-}$ ), total ESP variance (σ_tot_^2^), degree of charge balance (ν), molecular polarity index, and the fraction of polar surface area. Formal definitions of these descriptors are available in our previous work.^41,75–77^ Within the set of molecules evaluated here, these quantities consistently indicate that NVB exhibits higher propensity for self-association driven by electrostatic interactions, as reflected by the values obtained for νσ_tot_^2^.

From the MD trajectories, RDFs were computed within the *xy*-plane, which is parallel to the PS substrate, for NVB–NVB pairs to quantify NVB self-association within the adsorbed film (Figure 5c). The pronounced RDF peaks at short distances indicate strong lateral aggregation of NVB molecules across the PS surface, overall consistent with the ESP maps. The interaction between PS and NVB was further assessed by calculating the average Coulombic (*E*_coul_^NVB‑PS^ and *E*_coul_^NVB–NVB^) and Lennard–Jones (*E*_LJ_^NVB‑PS^ and *E*_LJ_^NVB–NVB^) interaction energies. The simulations predicted significantly stronger electrostatic interactions between NVB molecules than between NVB and PS (*E*_coul_^NVB–NVB^ = −772.70 ± 20.06 kJ/mol vs *E*_coul_^NVB‑PS^ = −28.53 ± 1.9 kJ/mol, respectively), whereas the Lennard–Jones interactions were comparable (*E*_LJ_^NVB–NVB^ = −75.14 ± 4.10 kJ/mol vs *E*_LJ_^NVB‑PS^ = −110.64 ± 3.62 kJ/mol, respectively). Overall, these computational data indicate a preference for NVB–NVB self-association on the PS surface. For completeness, it should be mentioned that these interaction energies are computed per NVB molecule. Importantly, these interactions are comparative energetic descriptors and should not be interpreted as adsorption free energies.

To further characterize the configurations adopted by NVB molecules at the solid–liquid interface, Figure 5d shows the orientational distribution of NVB molecules in contact with PS. The orientation is expressed as |cos θ|, where θ is the angle between a selected axis connecting two aromatic regions of NVB and the local PS surface normal. The enrichment at low |cos θ|, relative to the uniform distribution expected for isotropic orientations, indicates that the inter-aromatic axis preferentially lies approximately parallel to the PS interface. Nevertheless, the broad distribution suggests that NVB adopts heterogeneous, tilted configurations rather than a single adsorption geometry. It is worth highlighting that this descriptor represents only the selected inter-aromatic axis and should not be interpreted as the orientation of the entire NVB molecule nor that of all its aromatic rings; in fact, NVB contains an additional aromatic ring and flexible non-aromatic groups that may adopt different orientations, as indicated by visualization of the simulation snapshots.

While the adsorbed amount observed experimentally cannot be directly compared against studies using rougher microplastic particles, prior work consistently shows that hydrophobic and aromatic interactions govern the antibiotic-polystyrene affinity. MD simulations provide a way to probe these interactions at low surface densities at the temperature and pressure used in the experiments shown in Figure 4. The MD results show that, at the conditions probed, NVB forms a compact and apparently stable, within the length of the simulations, film on PS. Within these films, NVB is more likely to form aggregates than to spread uniformly on the PS surface. Dispersive LJ interactions between PS and NVB molecules contribute to stabilize NVB adsorption on PS as compared to Coulombic interactions. This observation is consistent with literature observations, both from electronic structure density functional theory and molecular simulations, indicating that interactions between the aromatic rings of complex molecules such as proteins and polymers and the PS substrate might play a role in the association between these molecules and the plastic substrate.^78,79^

### 3.2 Adsorption of Surfactants on NVB Films on Polystyrene

Sequential experiments were conducted to probe the potential competition between surfactants and NVB molecules for access to the PS surface. In these experiments, the NVB film was first formed on the PS substrate, and then surfactant solutions were flowed through the QCM-D measuring chamber. Because surfactants are frequently used in detergents, one might expect that exposing the antibiotic films to surfactant solutions may lead to antibiotic removal from the substrate. The results, shown in Figure 6 (Figure 6a adsorbed amounts, Figure 6b dissipation), tell a different story—in short, surfactants co-adsorb within or adsorb on top of the NVB film.

**Figure 6:**
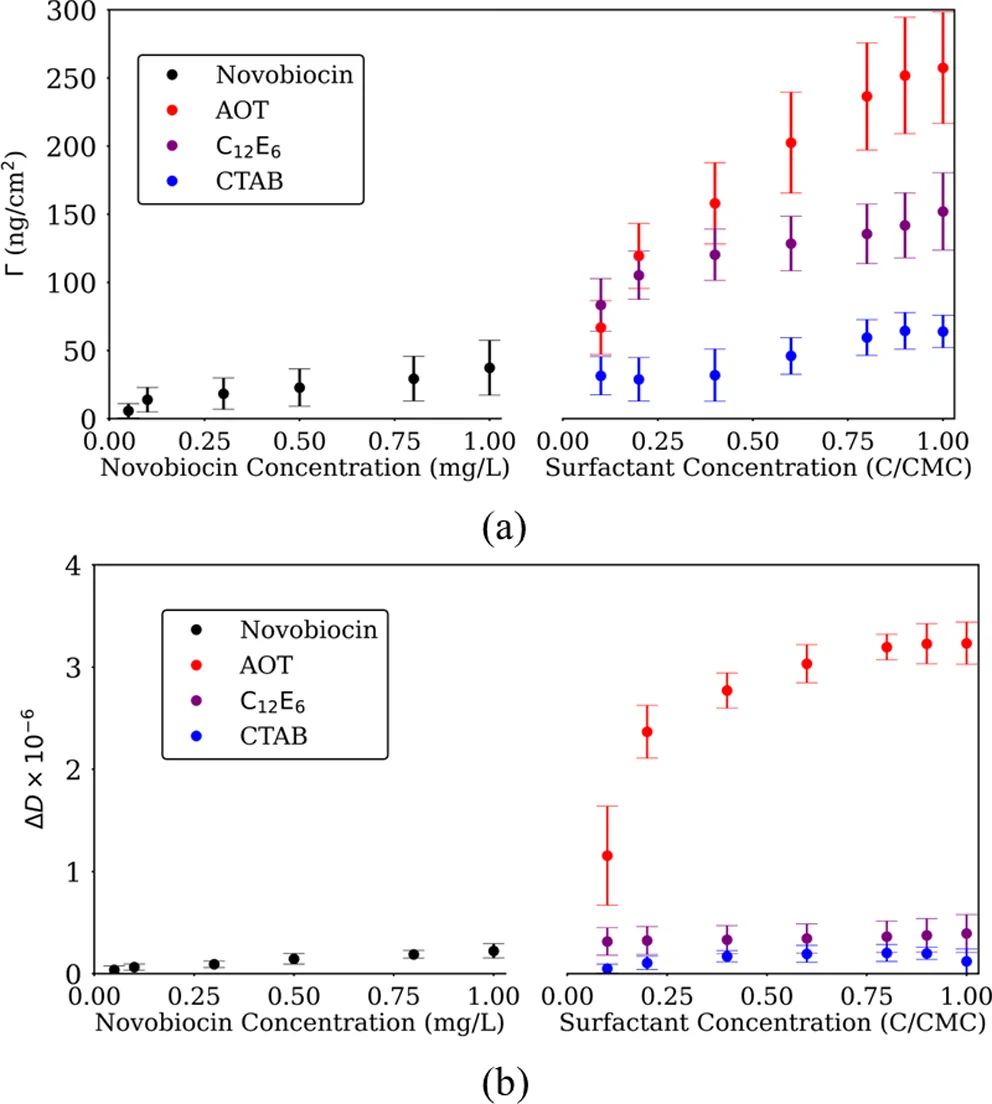
(a) Experimental adsorption isotherms obtained by first exposing the substrate to Novobiocin (NVB) aqueous systems at increasing concentration, followed by aqueous surfactant solutions and (b) the change of dissipation measured during the same experiments. The Sauerbrey equation was used to estimate the adsorbed mass for all systems considered. The experiments were conducted at 298 K.

In more detail, it can be seen from Figure 6 that when the substrate was first partially covered by NVB (the maximum amount adsorbed is estimated at 27 ng/cm^2^), and then exposed to aqueous surfactant solutions, the adsorbed amount continued to increase, reaching a plateau at concentrations above the surfactants CMC (plateau not clearly shown on the graph). For consistency, all three adsorption isotherms, including the one obtained for the NVB-AOT system, were modeled using the Sauerbrey equation. The small dissipation for most data suggests this interpretation is reliable for the comparative purposes of the present work. Although it is recognized that large dissipation values observed for the system containing AOT requires fitting using the Kelvin–Voigt model, applying this model to all systems is not recommended given the wide range in dissipations observed for the various systems (i.e., see Figure 6b). The raw data for the AOT isotherm reported in our previous work^41^ was reanalyzed here using the Saurbrey model to compare the estimated adsorbed mass obtained using the two models; the results are shown in Supporting Information, Figure S1. The maximum reported AOT adsorbed mass reduced from 670 ng/cm^2^ using the Kelvin–Voigt model to ∼250 ng/cm^2^ using the Sauerbrey model. The change in mass estimated by the Sauerbrey model indicated a lower bound of the true adsorbed mass for systems containing AOT; nevertheless, adsorption differences among the three surfactants can be compared using Figure 6a.

Figure 6a shows that AOT exhibited the highest mass adsorbed, followed by C_12_E_6_, and then CTAB on the PS substrate covered by NVB film. The trend reflects what was observed for the pure surfactant isotherms when NVB was not present in the system. However, the total amount of mass adsorbed at a surfactant concentration of 1 C/CMC is larger in Figure 6a than when the surfactants adsorbed on the PS substrate with no NVB film. For instance, at 1 C/CMC, C_12_E_6_ yields a film on PS with a mass of ∼120 ng/cm^2^, whereas the amount adsorbed when NVB is pre-adsorbed (Figure 6a) is ∼150 ng/cm^2^. The low dissipation of the NVB, CTAB, and C_12_E_6_ films indicates the formation of rigid layers with a limited contribution to the measured adsorption amount due to adsorbed water. Thus, the mass changes in these systems are attributed primarily to changes in the amounts of NVB and surfactant adsorbed, although their individual contributions cannot be identified by QCM-D data alone. In contrast, the pronounced increase in dissipation even at low AOT concentrations indicates the formation of hydrated, viscoelastic films containing AOT. The simultaneous increase in mass and dissipation is therefore consistent with AOT adsorption onto the pre-adsorbed NVB film. These results suggest that surfactants co-adsorb with NVB. Although it is possible that the surfactants remove some NVB, the results suggest that most of the pre-adsorbed NVB remains on PS.

AFM characterization of the pristine polystyrene sensors (see Figure 2) highlights the presence of nanotips with diameters ranging from 20 to 80 nm. Both surfactants and NVB adsorption are affected by the presence of pores and roughness on the substrate. Literature results show that a greater amount of antibiotics adsorbs on weathered microplastics, as more active sites become available for antibiotics to adsorb onto as the plastic surface degrades.^69^ For surfactants, however, the effect may be different: even though nanotips could provide additional regions for adsorption, adsorption at edges has been reported to be reduced compared to adsorption on flat surfaces.^80^

To complement experimental observations and visualize molecular differences in the structure of adsorbed antibiotic vs surfactant films, Figure 7a shows the density profiles along the *z*-direction, perpendicular to the PS substrate, for the MD simulated system containing C_12_E_6_ and NVB. The simulations confirm that NVB co-adsorbs with C_12_E_6_ on PS. The density profile for C_12_E_6_ is somewhat broader than that for NVB, indicating that the surfactant is well hydrated, with portions of its molecule extending into water. NVB maintains a density peak like the one observed for NVB on PS with no surfactant (panel a of Figure 5), suggesting that the nonionic surfactant does not disrupt the NVB assembled aggregates.

**Figure 7:**
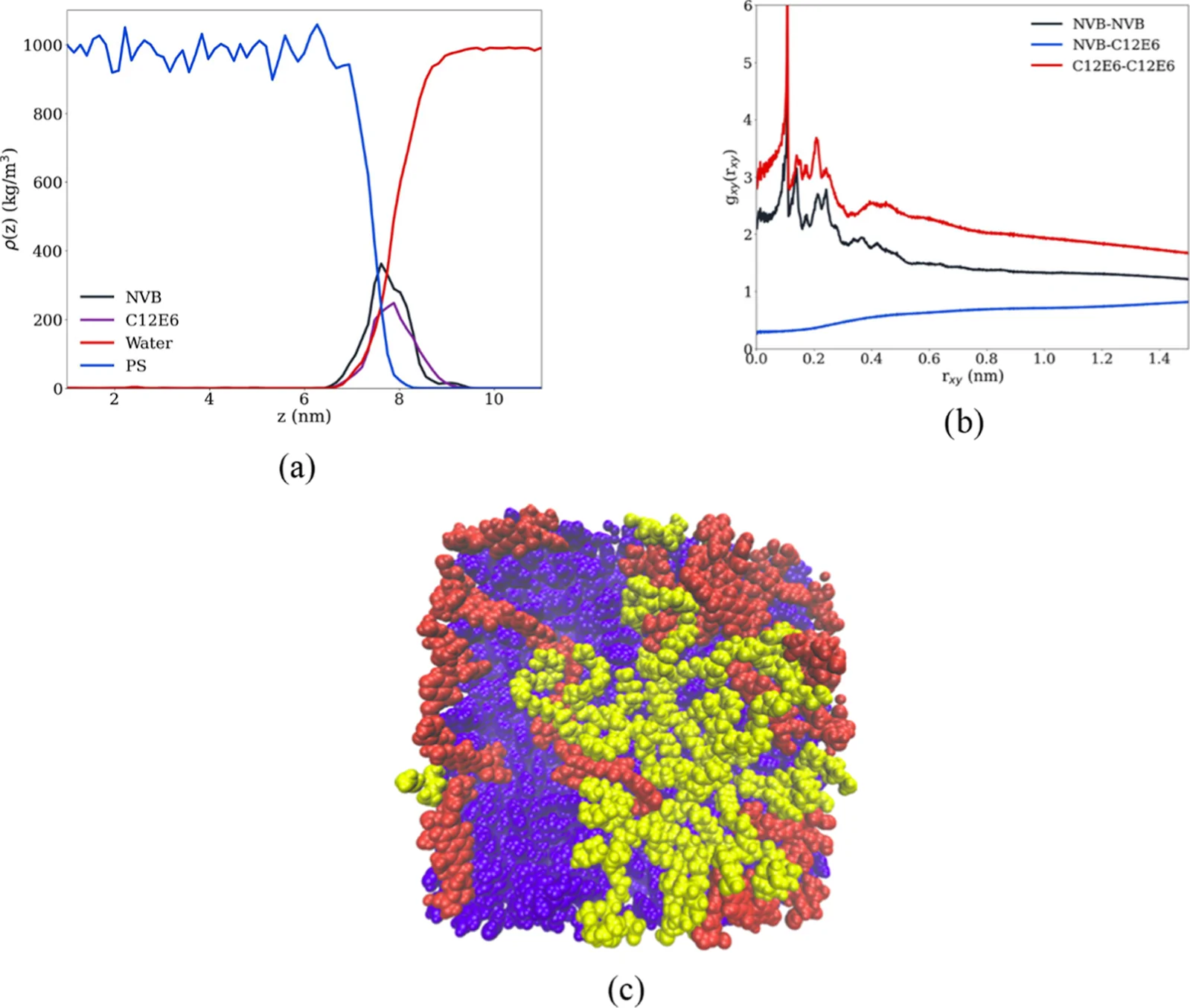
(a) Density profiles along the *z*-direction, perpendicular to the PS substrate, for the PS/NVB/water system in the presence of C_12_E_6_. (b) In-plane radial distribution functions (*g*_*xy*_(*r*)) between NVB and C_12_E_6_ adsorbed on PS, within a plane parallel to the substrate. (c) Representative simulation snapshot, top views of the PS substrate, illustrating the properties of the same system on the PS substrate. Color scheme: blue—PS; yellow—C_12_E_6_; red—NVB.

The results just described are similar to those obtained for NVB with CTAB, which are shown in Supporting Information for brevity (Figure S2a). However, NVB density has a more monomodal distribution in the presence of CTAB than in its absence, suggesting that the cationic surfactant may enhance NVB adsorption. Of note, density profiles indicate that CTAB forms a distinct interfacial layer on top of PS. When AOT is present (Figure S2b), density profiles suggest co-adsorption of AOT with NVB.

Based on the density profiles shown in Figures 5a, 7a, and S2, our simulations predict that although the surfactants impact NVB distribution, they do not significantly disrupt NVB adsorption, which is consistent with the experiments shown in Figure 4. To further quantify the MD results, radial distribution functions (RDFs) in the plane parallel to PS were calculated between NVB and adsorbed surfactants. The results shown in Figure 7b are for the system containing C_12_E_6_. The sharp RDF peaks found at short distances in both C_12_E_6_–C_12_E_6_ and NVB–NVB datasets indicate strong self-association. In contrast, the low-intensity NVB-C_12_E_6_ RDF peaks reflect weak interactions between NVB and the surfactant. This observation is qualitatively supported by the simulation snapshot in Figure 7c, which illustrates pronounced clustering between like molecules, i.e., surfactants with surfactants, and NVB with NVB. Similar results, shown in Figure S3 of the Supporting Information, are obtained for the other systems studied.

Finally, confirmation of molecular adsorption was obtained by quantifying the number of contacts between PS60 and NVB across the evaluated scenarios. Figure S4 shows that the number of NVB-PS heavy-atom contacts remains stationary between 200 and 500 ns for all systems, with no evident drift. In the absence of surfactant, NVB achieves the largest number of contacts with PS. The addition of AOT, C_12_E_6_, or CTAB reduces the extent of direct NVB-PS contact, indicating that the surfactants modify the interfacial organization of the adsorbed NVB layer. Nevertheless, a substantial number of NVB-PS contacts persist throughout all trajectories, supporting the conclusion that the surfactants do not completely displace NVB from the PS surface within the simulated time scale. Among the surfactant-containing systems, CTAB retains slightly more NVB-PS contacts than AOT and C_12_E_6_, although all three exhibit broadly similar behavior.

### 3.3 Adsorption of the NVB Solution onto Surfactant Films

To probe the strength of NVB-PS interactions, experimental isotherms were measured where the surfactant films were first formed on the substrate and then these films were exposed to aqueous solutions containing NVB at increasing concentrations, with results shown in Figure 8. Aqueous NVB solutions appear to partially remove the surfactant films; details are however surfactant specific. For example, the AOT film shows a significant reduction in dissipation (Figure 8b), consistent with a film that becomes more rigid upon exposure to NVB. The data suggest that even 0.1 mg/L of NVB induces substantial changes within the AOT film, reducing the dissipation to levels observed at low bulk AOT concentrations/high NVB concentrations while also reducing significantly the total adsorbed mass. The final adsorbed amount is slightly larger than what was observed for NVB alone, which is consistent with some remaining AOT molecules co-adsorbed with NVB.

**Figure 8:**
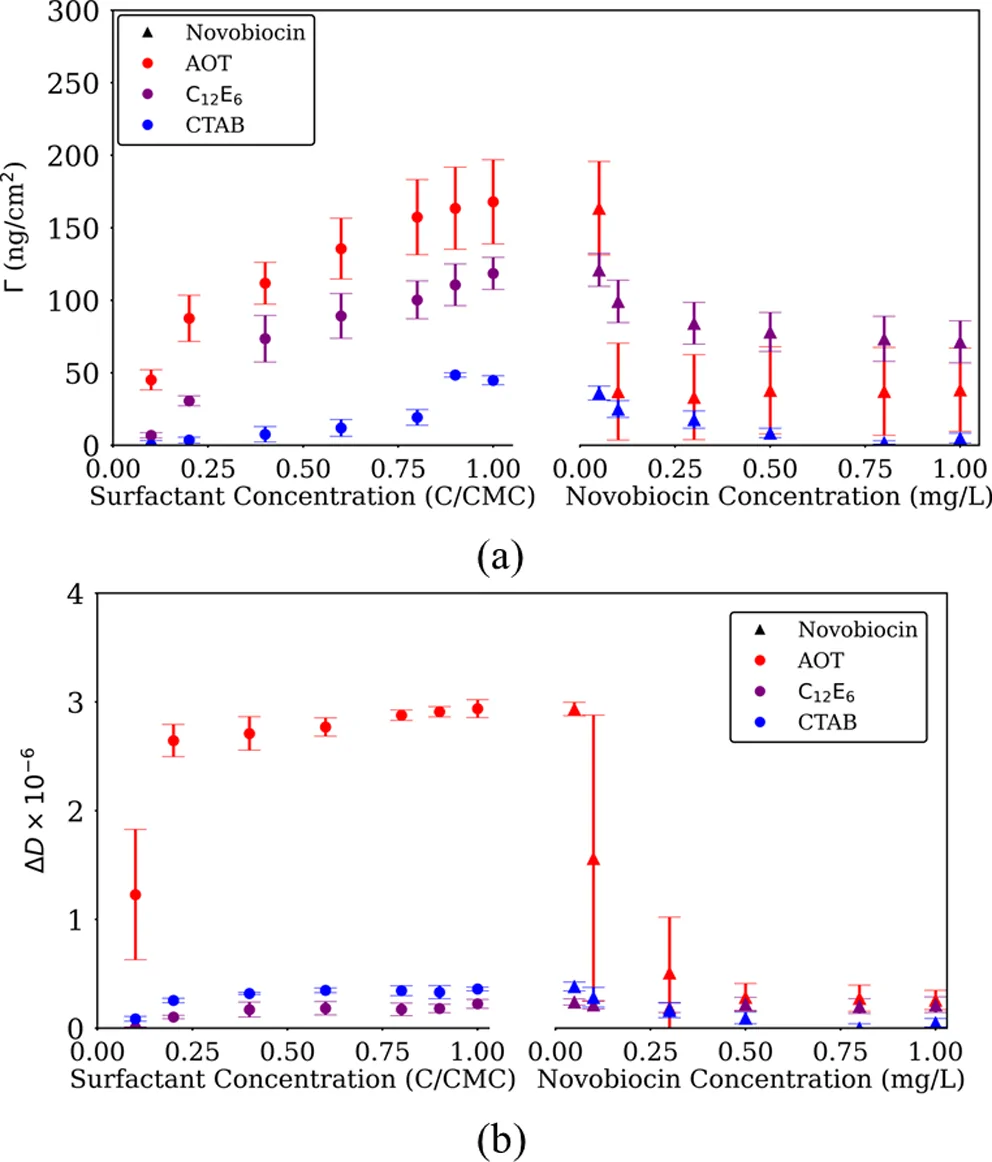
(a) Experimental adsorption isotherms obtained by first exposing the PS substrate to aqueous surfactant solution at increasing concentration, followed by NVB aqueous systems and (b) the change of dissipation. The Sauerbrey equation was used to estimate the adsorbed mass in all cases. The experiments were conducted at 298 K.

In contrast, when the C_12_E_6_ film is pre-adsorbed, the total mass adsorbed gradually decreases as the bulk NVB concentration increases, reaching a plateau at 70 ng/cm^2^ at 0.3 mg/L of NVB. This adsorbed amount (i.e., 70 ng/cm^2^) is higher than what was measured when PS is exposed only to NVB (cf. Figure 6a). This result is consistent with NVB partially displacing some, but not all the C_12_E_6_ pre-adsorbed on PS.

Finally, when the CTAB film was exposed to aqueous NVB, the adsorbed mass decreased as the bulk NVB concentration increased, with a slight increase observed only after 1 mg/L of NVB was introduced. In this case, the results suggest that the NVB solution completely removed the pre-adsorbed film or the amount of CTAB + NVB adsorbed matched that of pure NVB adsorption. Overall, these results suggest that some of the surfactants are removed by the NVB solution (perhaps all in the CTAB case) while some NVB adsorbs always leading to films with low viscoelasticity.

To complement the experimental results just discussed, Figure 9 presents results from molecular dynamics simulations illustrating density profiles along the direction perpendicular to the PS substrate for simulated systems in which the surfactants were first adsorbed at the PS/water interface, followed by the introduction of NVB. The results reveal that the surfactants remain closer to PS compared to NVB, which adsorbs over the pre-existing surfactant layer. This suggests that the order of adsorption impacts the interfacial organization. Although current computational limitations prevent us from assessing whether NVB can remove pre-adsorbed surfactants to engage with the PS substrate, the results clearly show that NVB adsorbs at the PS/water interface even in the presence of pre-adsorbed surfactants.

**Figure 9:**
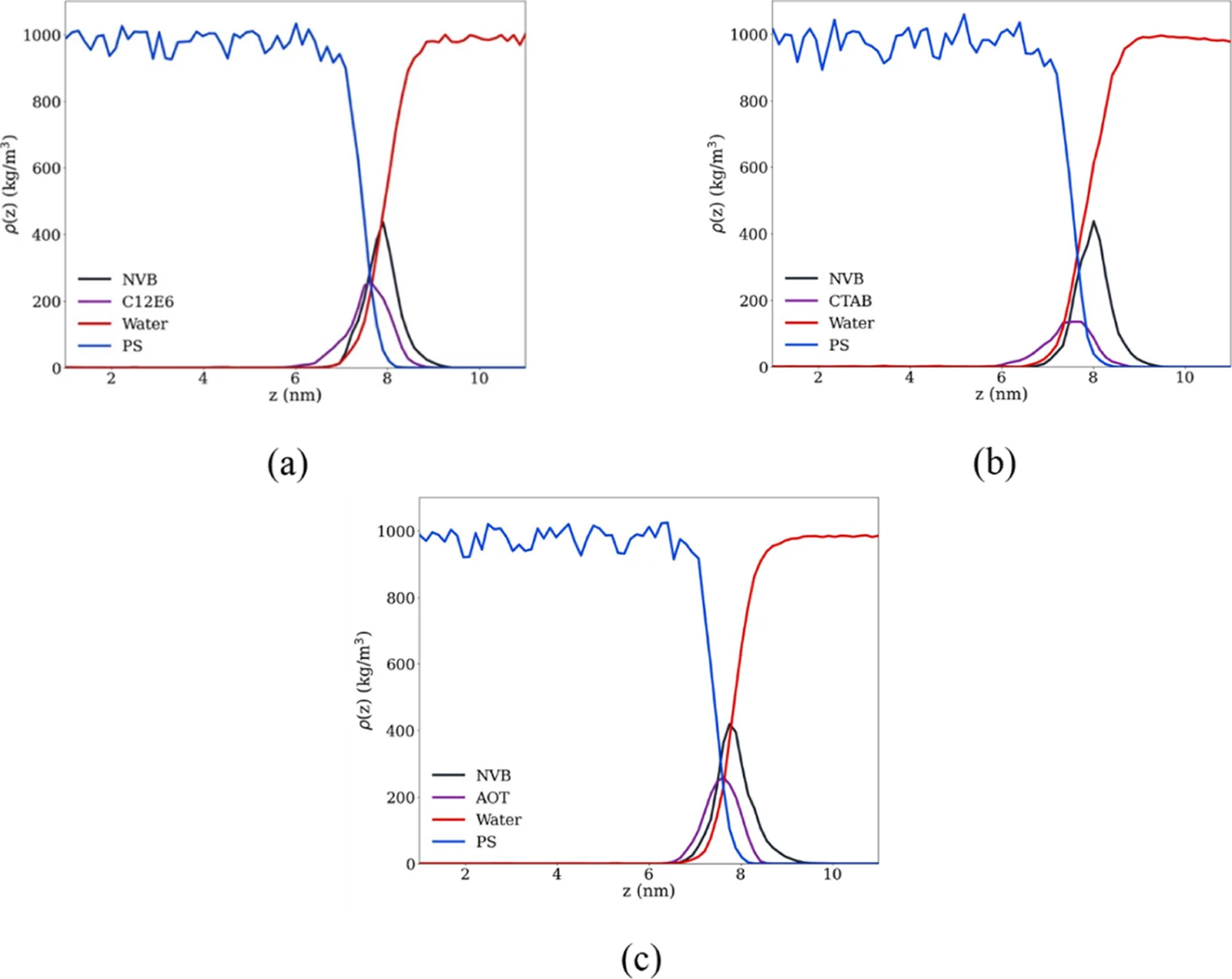
Simulated density profiles along the *z* direction, perpendicular to the PS substrate, for the systems simulated using molecular dynamics. In these simulations, surfactants were first adsorbed onto the PS/water interface; when the systems were equilibrated, NVB molecules were inserted at the interface. Results are shown for (a) C_12_E_6_, (b) CTAB, and (c) AOT. The simulations were conducted at 298 K.

## 4 Conclusions

Results presented in this work show that the antibiotic NVB adsorb from water on PS substrates. NVB has strong interactions with PS, although NVB molecules preferentially self-associate at the surface, forming clusters rather than spreading uniformly across the PS interface. Experimental adsorption isotherms show that when NVB films are pre-adsorbed on the substrate, surfactants either co-adsorb within it, or adsorb on the antibiotic film. The surfactants considered are common commercial surfactants belonging to cationic, nonionic, and anionic families, suggesting that the behavior might be generalizable to other surfactants as well. It is, however, expected that the results strongly depend on the plastic substrate and on aging. The experimental data also shows that aqueous solutions containing NVB but no surfactant can partially remove pre-adsorbed surfactant films. The details depend on the surfactant. NVB is found to co-adsorb with AOT and C_12_E_6_, while it likely removes CTAB. Molecular simulation results show that specific interactions between molecules, in particular those driven by partial charges and aromatic rings, are responsible for these differences.

While generalization is premature, these findings underscore the importance of understanding potential synergistic effects among different emergent contaminants. These results could nevertheless inform the design of effective wastewater treatment processes to remove plastics, microplastics, and antibiotics.

## Supplementary Material



## Data Availability

All data will be provided upon request.

## Notes

GIPF values calculated for novobiocin; simulated density profiles in the direction perpendicular to the PS substrate for selected compounds; selected radial distribution functions; data obtained to confirm equilibration of the simulated systems (PDF)
