# Supplementary material for: Co-Adsorption of Novobiocin and Three Common Surfactants on Polystyrene: Molecular Insights from Experiments and Simulations
Source: Langmuir. 2026 Sep 12;42(38):27874–87. doi: 10.1021/acs.langmuir.6c02613 (PMC13629672; doi:10.1021/acs.langmuir.6c02613)
Supplement: Supplementary file 1 [file la6c02613_si_001.pdf]

Supplementary Information

Co-Adsorption of Novobiocin and Three Common Surfactants on Polystyrene: Molecular Insights from Experiments and Simulations

D. S. Barrios,[a] G. D. Barbosa,[a] S. Razavi,[a,b] H. I. Zgurskaya,[b,c] B.P. Grady,[a,b] A. Striolo[a,b,1]

[a] *School of Sustainable Chemical, Biological and Materials Engineering, University of Oklahoma, Norman, OK 73019, USA*

[b] *Materials Science and Engineering Program, The University of Oklahoma, Norman, OK 73019, United States*

[c] *Department of Chemistry and Biochemistry, The University of Oklahoma, Norman, OK 73019, United States*

- GIPF descriptors from DFT calculations are shown in Table S1.
- AOT adsorption isotherm originally analyzed using the Kelvin–Voigt model in Ref. 1 and reanalyzed isotherm using the Sauerbrey model are shown in Figure S1.
- Density profiles in the direction perpendicular to the PS substrate for the PS/NVB/water system with CTAB and AOT are shown in Figure S2.
- In-plane RDFs, *g(r)*, for the PS/NVB/water system with CTAB and AOT, calculated within planes parallel to the PS substrate, are shown in Figure S3.

[1] Corresponding author email: astriolo@ou.edu

Table S1: General Interaction Property Functions (GIPF) of selected compounds, as predicted by density functional theory and calculated using the molecular electron density isosurface 0.00100 e/Bohr^3.

Reference values for AOT, CTAB, and $C_{12}E_6$ were taken from our recent work.[1]

Comp.	V_m	V_{min}	V_{max}	SA	$\bar{V}^+$	$\bar{V}^-$	$\nu\sigma_{tot}^2$	MPI	Polar SA (%)
NOVO	715.5	-72.4	126.2	561.0	23.9	-20.9	178.8	22.6	70.8
AOT	566.3	-50.4	108.0	423.9	13.0	-14.9	117.4	13.7	49.8
CTAB	515.2	-54.4	45.7	392.0	19.26	16.2	115.2	16.2	47.3
$C_{12}E_6$	652.3	-35.7	14.1	484.9	5.6	6.31	11.2	6.3	19.5

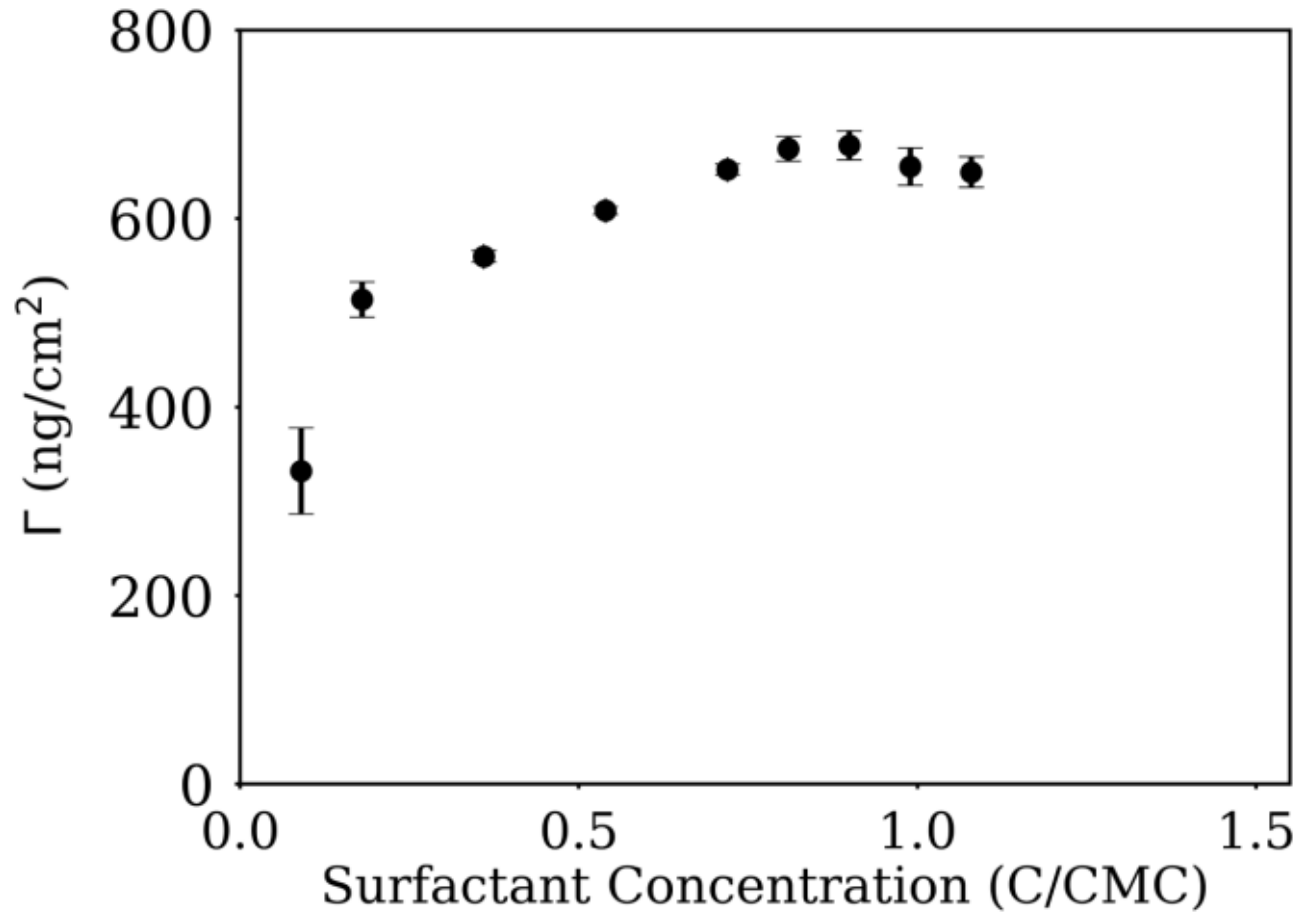

(a)

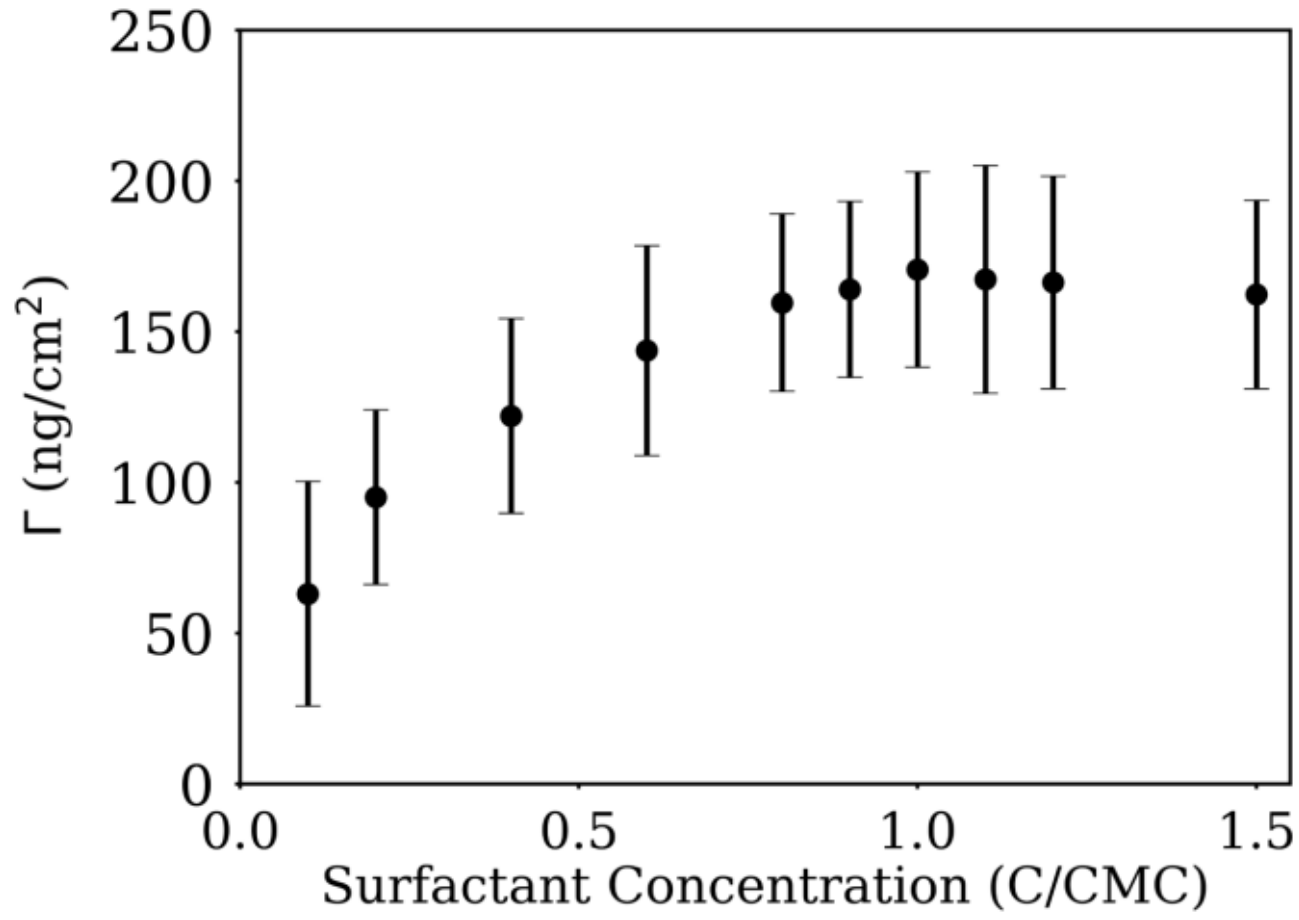

(b)

Figure S1: (a) Pure AOT adsorption isotherm originally analysed in Ref. 1 using the Kelvin–Voigt model and (b) reanalysed with the Sauerbrey model for consistent comparison.

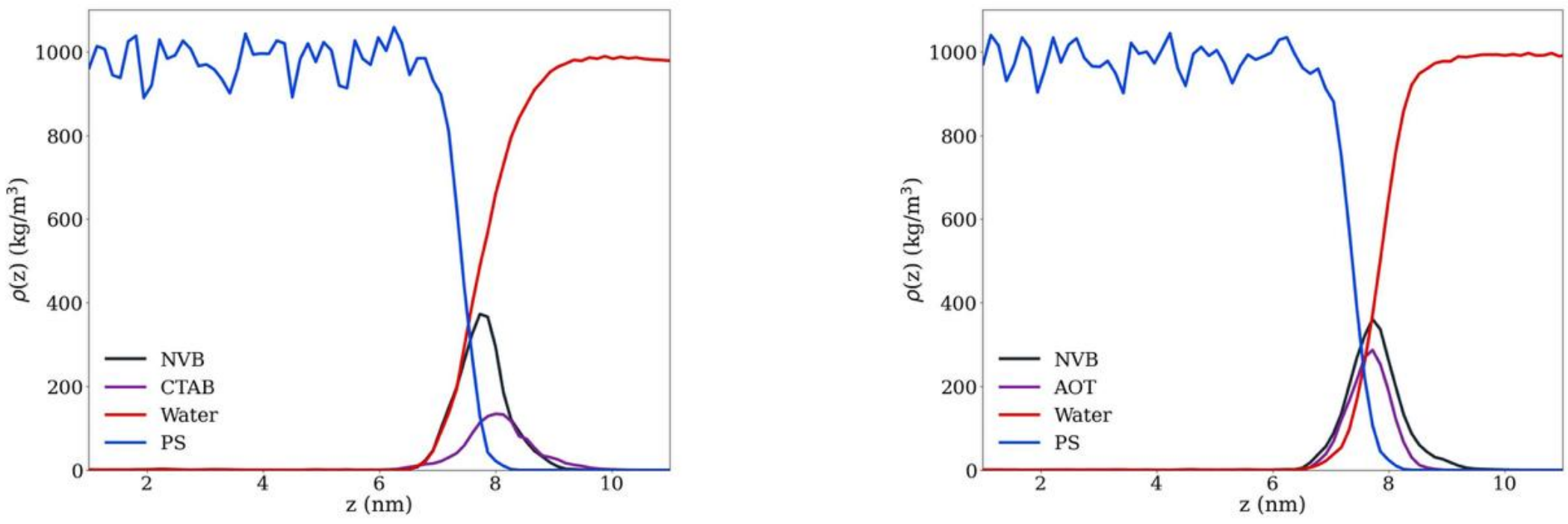

(a) (b)

Figure S2: Density profiles along the z-direction, perpendicular to the PS substrate, for the PS/NVB/water system in the presence of different surfactants as obtained from molecular dynamics simulations: (a) CTAB, and (b) AOT.

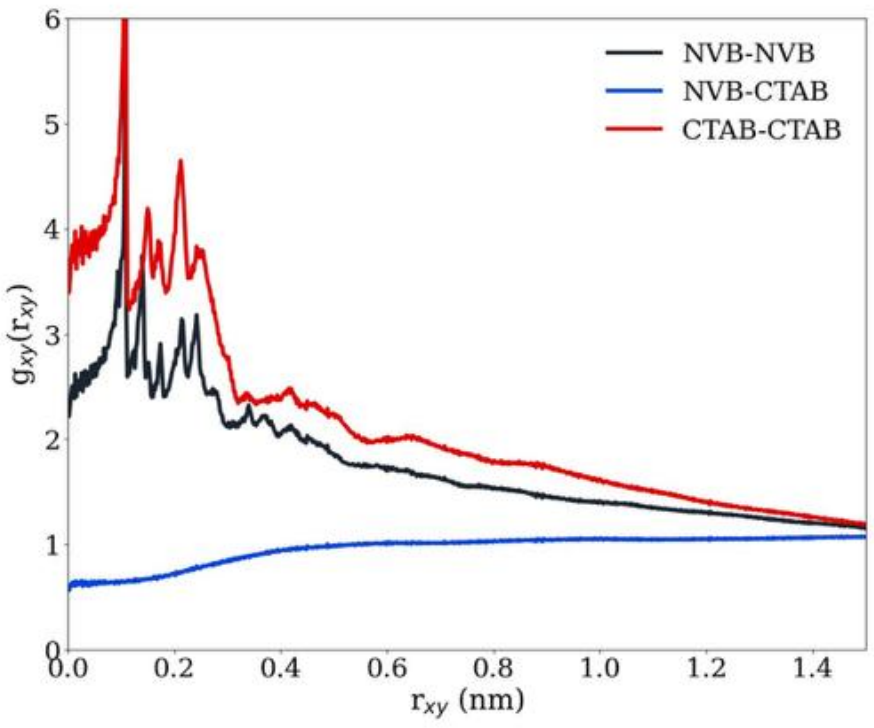

(a)

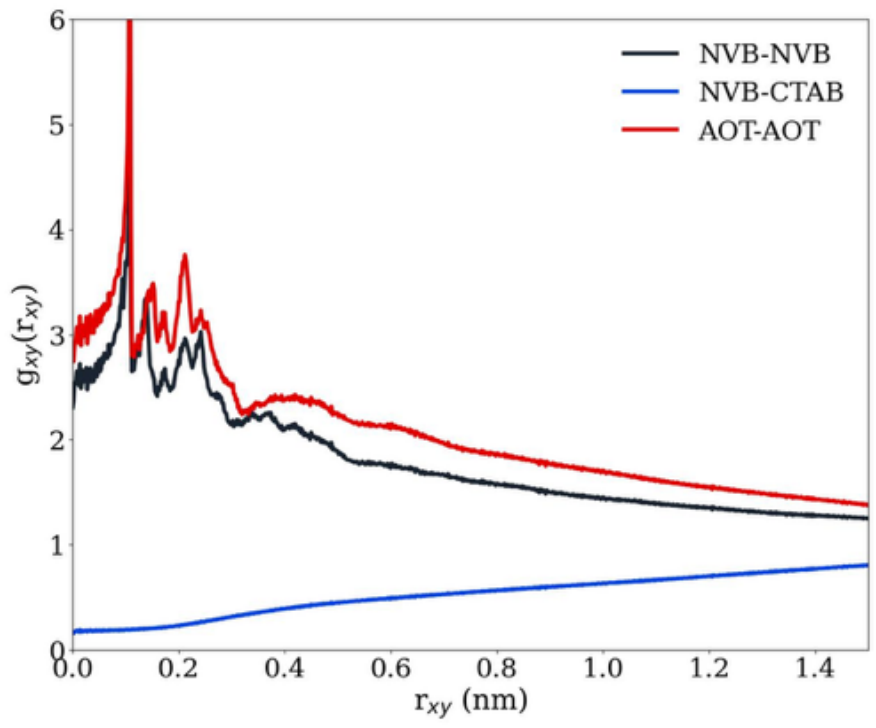

(b)

Figure S3: Radial distribution functions (RDFs) g(r) in the xy-plane, parallel to the PS substrate, for the PS/NVB/water system in the presence of different surfactants as obtained from molecular dynamics simulations: (a) CTAB, and (b) AOT.

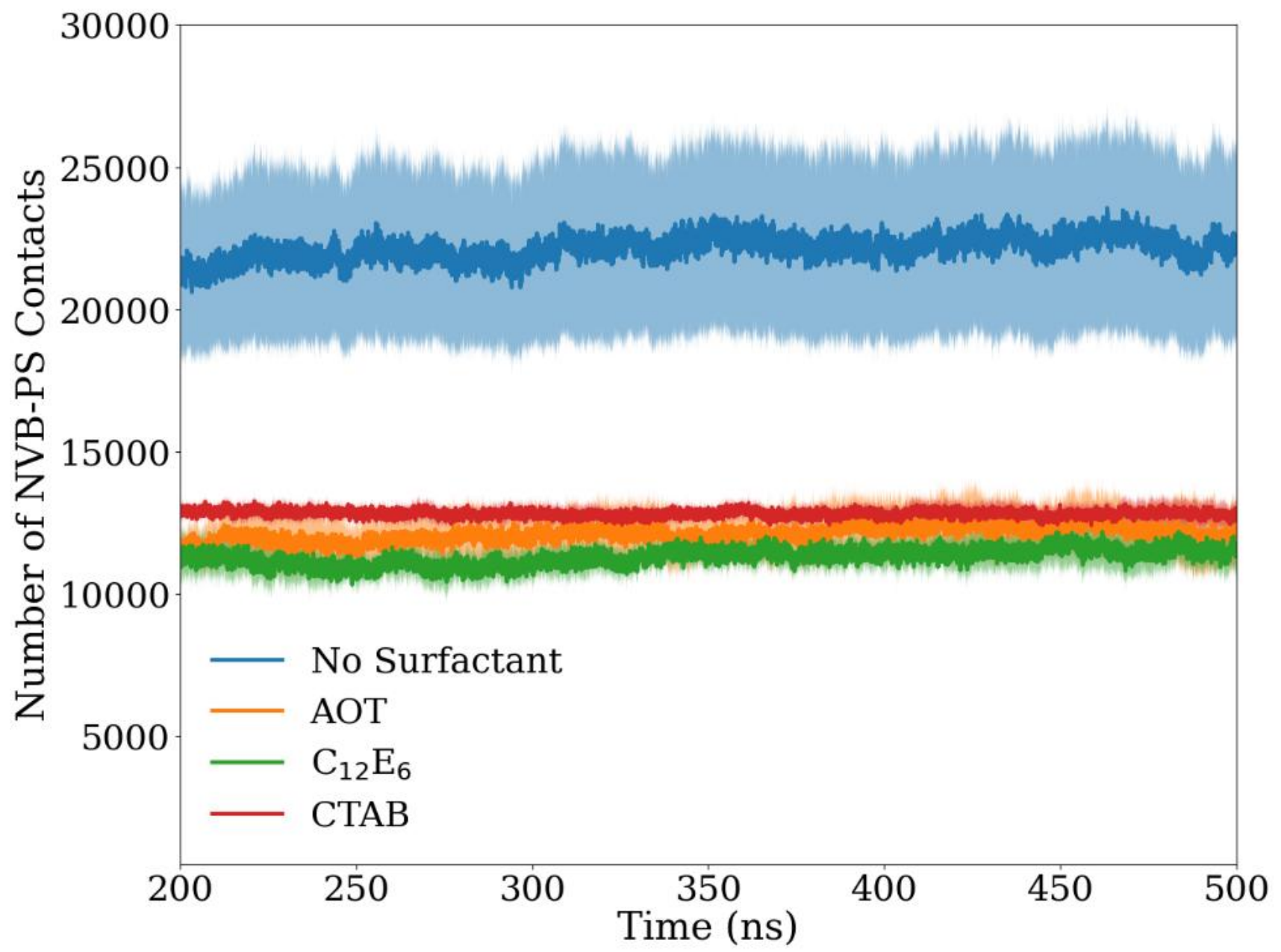

Figure S4. Time evolution of the number of NVB–PS heavy-atom contacts for systems in which NVB was initially adsorbed on the PS surface, in the absence (results identified as 'No Surfactant') and presence of AOT, $C_{12}E_6$, or CTAB. A contact was defined when an NVB heavy atom and a PS heavy atom were separated by less than 0.60 nm. Solid lines represent the mean obtained from three independent replicas, and the shaded regions indicate the corresponding standard deviation.

References

(1) Barrios, D. S.; Barbosa, G. D.; Razavi, S.; Grady, B. P.; Striolo, A. What molecular and surface features control surfactant films on polystyrene? *Surfaces and Interfaces* **2026**, *91*. DOI: 10.1016/j.surfin.2026.109217.
